# Effects of compatibility of *Clostridium butyricum* and *Bacillus subtilis* on growth performance, lipid metabolism, antioxidant status and cecal microflora of broilers during the starter phase

**DOI:** 10.5713/ab.24.0132

**Published:** 2024-06-27

**Authors:** Xu Zhao, Jiarong Zhuang, Faling Zhang, Hongtao Li, Juan Yu, Chengli Wang, Tengjiao Lv, Qingzhen Li, Jimei Zhang

**Affiliations:** 1 College of Agriculture and Forestry Science, Linyi University, Linyi 276000, China; 2 Linyi Backbone Biotechnology Co., Ltd., Linyi 276036, China; 3 Shandong Lonct Enzymes Co., Ltd., Linyi 276400, China

**Keywords:** *Bacillus subtilis*, Broiler, Cecal Microflora, *Clostridium butyricum*, Growth, Lipid Metabolism

## Abstract

**Objective:**

This study aimed to determine the effects of compatibility of *Clostridium butyricum* and *Bacillus subtilis* on growth performance, lipid metabolism, antioxidant status and cecal microflora of broilers during the starter phase.

**Methods:**

A total of 600 1-day-old Ross 308 broilers were randomly divided into two groups with six replicates in each group. Chickens in the control group were fed a basal diet, while chickens in the experimental group were fed a diet supplemented with 2×10^8^ colony forming units (CFU)/kg of *C. butyricum* and 1×10^9^ CFU/kg of *B. subtilis*. The experimental period was 21 days.

**Results:**

Addition of *C. butyricum* and *B. subtilis* significantly increased (p<0.05) the body weight and liver nicotinamide adenine dinucleotide phosphate-malic enzyme (NADP-ME) activity of broilers, enhanced (p<0.05) the average daily gain and average daily feed intake of broilers. However, the addition of *C. butyricum* and *B. subtilis* did not significantly affect the concentrations of triglyceride and total cholesterol in the serum, the activities of fatty acid synthase and acetyl–CoA carboxylase in the liver, the total antioxidant capacity, glutathione peroxidase activity and malondialdehyde content in the serum and liver. Besides, microbial analysis revealed that supplementation of *C. butyricum* and *B. subtilis* increased (p<0.05) the abundance of *Firmicutes* such as *CHKCI001* and *Faecalibacterium*, decreased (p<0.05) the abundance of *Bacteroidota* such as *Bacteroides* and *Alistipes*. Spearman correlation analysis confirmed that the above cecal microbiota were closely related to the growth performance of broilers (p<0.05). In addition, simultaneous supplementation of *C. butyricum* and *B. subtilis* significant affected (p<0.05) 33 different functional pathways such as lipid metabolism and carbohydrate metabolism. This explains the phenomenon of increased growth performance and liver NADP-ME activity in the probiotics group.

**Conclusion:**

The compatibility of *C. butyricum* and *B. subtilis* could improve the growth of broilers during the starter phase by changing the cecal microflora.

## INTRODUCTION

Poultry’s digestive tract contains many microorganisms that play an important role in maintaining the relative stability of intestinal environment and enhancing digestion and absorption [1]. In recent years, studies have shown that some changes in poultry intestinal microorganisms can have an impact on the antioxidant capacity of poultry through the nuclear factor erythroid 2-related factor 2 (Nrf2) signaling pathway [2]. In addition, the relative abundance of microorganisms in the poultry intestine, especially the dominant phyla *Firmicutes* and *Bacteroidetes*, can be linked to the body weight (BW) and lipid metabolism of poultry through the circulating factor angiopoietin-like protein 4 (ANGPTL4) [3,4]. Therefore, improving the intestinal microorganisms of poultry is of great significance for improving their antioxidant capacity and growth performance. At present, there are many ways to change the intestinal microorganisms of poultry, among which exogenous probiotics is one of the more effective methods.

*Clostridium butyricum* is an anaerobic gram-positive bacterium, which is characterized by production of butyric acid [5]. *Bacillus subtilis* is aerobic in nature [6,7], but under anaerobic conditions, they can utilize nitrate or nitrite as the terminal electron acceptor to facilitate anaerobic respiration [8]. These two probiotics, which belong to *Firmicutes*, have been approved as feed additives for broilers (Announcement No. 2045 of the Ministry of Agriculture of the People’s Republic of China). Since both *C. butyricum* and *B. subtilis* can produce spores, the two probiotics have strong resistance to the external environment, and have strong tolerance to high temperature and high pressure in the feed processing process, as well as to gastric acid and bile acid of animals [7,9]. Studies have shown that *C. butyricum* and *B. subtilis* can improve the growth performance and modify the intestinal microbial composition of broilers [3,10], but there are few studies on the compatibility of these two probiotics in broilers production. As mentioned above, *B. subtilis* is an aerobic bacterium, which can consume oxygen in the intestinal tract after being eaten by broilers, which will provide powerful conditions for the reproduction and growth of *C. butyricum*, so the compatibility of *C. butyricum* and *B. subtilis* will theoretically play a synergistic role. The starter phase of broilers is an important period for the gradual establishment and improvement of the intestinal microflora of broilers. During this period, the intestinal microflora of broilers is often greatly affected by external factors [11,12]. Therefore, this study explored the effects of compatibility of *C. butyricum* and *B. subtilis* on growth performance, lipid metabolism, antioxidant status and cecal microflora of broilers during the starter phase.

## MATERIALS AND METHODS

### Animal care

The animal care and use protocol was approved by the Ethics Committee of Linyi University (Linyi, Shandong, China) (20200107).

### Birds and treatments

Six hundred healthy one-day-old Ross 308 broilers with similar BW (40.50±0.15; purchased from Shandong Minhe Animal Husbandry Co., Ltd., Shandong, China) were randomly divided into two groups with six replicates per group and 50 broilers per replicate. Broilers in the control group were fed the basal diet (composition and nutrient levels as shown in Table 1) formulated according to the nutritional recommendation of National Research Council [13]. Chickens in the experimental group were given the basal diet supplemented with 2×10^8^ colony forming units (CFU)/kg of *C. butyricum* and 1×10^9^ CFU/kg of *B. subtilis* (provided by Shandong Hongde Agriculture and Animal Husbandry Technology Co., Ltd., the added dosage of the two probiotics is the recommended dosage given by the provider based on previous practical experience, and the data are unpublished). The strains of *C. butyricum* and *B. subtilis* used in this study were *C. butyricum* HJCB998 (CGMCC 9386) and *B. subtilis* HJBA058 (CGMCC 9383), respectively. The diets for both groups were pellet form. The contents of crude protein, calcium, total phosphorus and amino acid in diet were determined according to China national standards GB/T 6432-2018, GB/T 6436-2018, GB/T 6437-2018, and GB/T 18246-2019, respectively. The experimental broilers were raised in three-layer cages with free access to feed and water ([100×80] cm^2^ floor space, 10 broilers in each cage), and raise according to the normal feeding and management mode. The temperature was maintained at 34°C±1°C in the first three days, and then decreased by 1°C every 2 to 3 days until the broilers were 21 days of age. The humidity was maintained at 60% to 65% in the first week and 55% to 60% thereafter. In the first two days, 24 hours of light were provided every day, followed by a schedule of 23 hours of light and 1 hour of darkness per day. The broilers used in this experiment were raised in Wangnong Breeding Co., Ltd. (Junan, Linyi, Shandong, China). The experimental period was 21 days.

### Sample collection

One healthy broiler (with a BW close to the average weight of the broilers in the replicate) from each replicate that had been fasting for 12 h was selected to take blood samples from the wing vein at the end of the experiment (the broilers was 21 days of age at this time). Serum was then separated for detection of serum lipids and antioxidant status related indicators. Afterwards, the same broilers were then euthanized by arterial exsanguination under deep anesthesia after feeding for 2 h. Liver samples from the same part on the left side were isolated and stored at −20°C for the detection of the lipid synthesis-related enzyme activities and the antioxidant status related indicators. The contents of the left cecum were collected in 7 mL sterilized centrifuge tubes and stored at −80°C after quick freezing with liquid nitrogen for detection of cecal microflora.

### Measurement of growth performance

During the experiment, the feed intake, initial and final BW and health status of the broilers in each replicate were recorded, and the average daily feed intake (ADFI), average daily gain (ADG), and feed conversion rate (FCR) of the control group and the experimental group were calculated.

### Determination of triglyceride and total cholesterol in the serum and antioxidant status in the serum and liver

The contents of triglyceride (TG) and total cholesterol (TC) in the serum, the total protein content in the liver, as well as the total antioxidant capacity (T-AOC), glutathione peroxidase (GSH-Px) activity, and malondialdehyde (MDA) content in the serum and liver were determined using the corresponding kits from Nanjing Jiancheng Bioengineering Institute (Nanjing, Jiangsu, China). Among these indicators, liver T-AOC and MDA content were measured using 10% liver tissue homogenate supernatant, liver GSH-Px activity was measured using 6.67% liver tissue homogenate supernatant, and liver total protein content was measured using 1% liver tissue homogenate supernatant. The serum GSH-Px activity was measured using 10-fold diluted serum, and the remaining serum-related indicators were measured using undiluted serum. The determination method was the same as the kit instruction.

### Lipid synthesis-related enzyme activity assays

The activities of fatty acid synthase (FAS), NADP-malic enzyme (NADP-ME), acetyl-CoA carboxylase (ACC) and content of total protein in the liver were determined by the corresponding kits produced by Suzhou Comin Biotechnology Co., Ltd. (Suzhou, Jiangsu, China). The preparation methods of liver tissue homogenates and the determination methods of the above indexes were consistent with the instructions of the corresponding kits.

### Cecal microflora assays

The cecal microflora was determined by 16S rDNA amplicon sequencing, and the library was paired-ended sequenced by Beijing Novogene Technology Co., Ltd. (Beijing, China) based on the illumina NovaSeq sequencing platform. The specific operation process was the same as that described by Li et al [14]. After sequencing, paired-end reads were processed by merging, data filtration and chimera removal to obtain the effective tags [15]. Then, the effective tags were denoised (using the DADA2 module in the QIIME2 software [QIIME2-202006 version]), and the sequences with abundance less than 5 were filtered out to obtain the Amplicon Sequence Variants (ASVs). Next, species annotation was performed on each ASV using the classify-sklearn module in the QIIME2 software (Silva Database), and according to the results of species annotation, the top 10 species with the highest abundance at the phylum and genus levels in each group were selected to generate a species relative abundance column cumulative graph to intuitively view the species with higher relative abundance and their proportions at the phylum and genus levels in each group of samples. Alpha diversity such as chao1, observed_otus, shannon and simpson was calculated with QIIME2 (using t-test). For beta diversity analysis, the principal co-ordinates analysis (PCoA) based on weighted UniFrac distances result was displayed using ade4 package and ggplot2 package in R software (Version 2.15.3). In addition, the microbial differences between the two groups were measured by linear discriminant analysis (LDA) effect size (LEfSe) (Version 1.0, https://huttenhower.sph.harvard.edu/galaxy/)(LDA>4, p<0.05) [16]. The correlation between growth performance and microbial species richness was analyzed using Spearman correlation analysis. The functional pathways prediction of cecal flora was inferred using Tax4Fun, the specific steps are consistent with those described by Cheng et al [17].

### Statistical analysis

The independent sample t-test was carried out on the data by SAS9.2 statistical software (SAS Institute, Inc., Cary, NC, USA). The results were expressed as mean±standard error. Statistical significance is defined when p values are less than 0.05. Five cages were considered as the experimental unit.

## RESULTS

### Growth performance

The BW, ADG, and ADFI of broiler chickens fed a diet supplemented with *C. butyricum* and *B. subtilis* were significantly higher (p<0.05) than those of the control group (Table 2). However, no difference was observed in FCR between control and *C. butyricum* and *B. subtilis*-supplemented group birds.

### Serum lipid

The contents of TG and TC in the serum of broilers in bacteria addition group were not significantly different from those in control group (Table 3).

### Lipid synthesis-related enzyme activity

The activity of NADP-ME in the liver of broilers in the probiotic supplemented group was significantly higher (p<0.05) than that in the control group, but the activities of FAS and ACC were not significantly different from those in the control group (Table 4).

### Antioxidant status

The indexes of T-AOC, GSH-Px, and MDA in the serum and liver of broilers supplemented with probiotics were not significantly different from those of the control group (Table 5).

### Diversity of cecal microbiota

#### Alpha diversity

The Shannon and Simpson index of broilers added with *C. butyricum* and *B. subtilis* were significantly lower (p<0.05) than those of the control group (Table 6). However, the Chao1 and Observed_otus of broilers added with *C. butyricum* and *B. subtilis* were not significantly different from those of the control group.

#### Beta diversity

**Figure 1:**
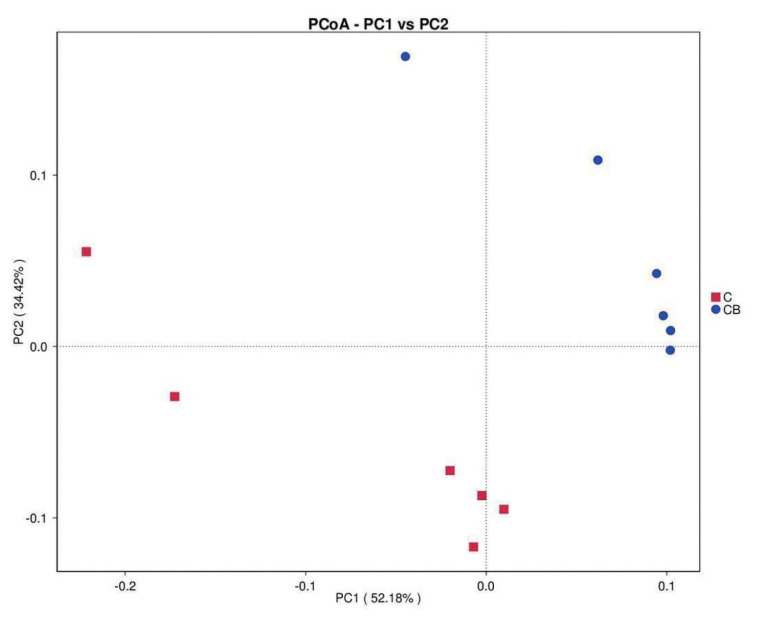
The principal co-ordinates analysis (PCoA) based on weighted UniFrac distances reflecting species beta diversity within and between groups. C, basal diet provided as control; CB, basal diet supplemented with 2×10^8^ CFU/kg of *C. butyricum* and 1×10^9^ CFU/kg of *B. subtilis*; CFU, colony forming units.

The PCoA based on weighted UniFrac distances revealed clear clustering of cecal microflora between the probiotic group and the control group (Figure 1).

### Variation in cecal microbiota composition

**Figure 2:**
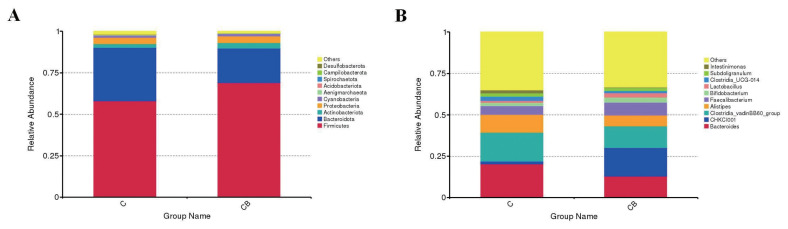
Composition of the top 10 cecal microflora of 21-day-old broilers. (A) Phylum level. (B) Genus level. Values are expressed as the means. C, basal diet provided as control; CB, basal diet supplemented with 2×10^8^ CFU/kg of *C. butyricum* and 1×10^9^ CFU/kg of *B. subtilis*; CFU, colony forming units.

*Firmicutes* and *Bacteroidota* were the dominant phyla in the cecal microbiota of 21-day-old broilers, accounting for nearly 90% of the phylum in the cecal microbiota (Figure 2A). *Bacteroides*, CHKCI001, *Clostridia* vadinBB60 group, *Alistipes*, and *Faecalibacterium* accounted for more than 50% of the genera in the cecal microbiota of 21-day-old broilers (Figure 2B).

**Figure 3:**
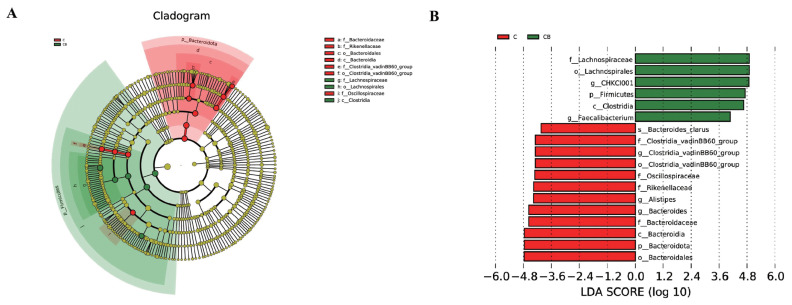
Linear discriminant analysis (LDA) effect size (LEfSe) analysis of the cecal microflora. (A) Taxonomic cladogram. From the inside to the outside, the circle is classified as phylum (p), class (c), order (o), family (f), genus (g), and species (s). The diameter of the small circle at each taxonomic level is proportional to its relative abundance. The small green circles (green legend) are the taxa enriched in group CB, the small red circles (red legend) are the taxa enriched in group C, and the small yellow circles are the taxa with no significant differences between the group CB and the group C. (B) LDA value distribution histogram (taxa with LDA score>4). C, basal diet provided as control; CB, basal diet supplemented with 2×10^8^ CFU/kg of *C. butyricum* and 1×10^9^ CFU/kg of *B. subtilis*.

The LEfSe analysis identified 18 biomarkers (Figure 3). Among them, six bacterial taxa were enriched in broilers in the *C. butyricum* and *B. subtilis*-supplemented group, including *Lachnospiraceae* (family), *Lachnospirales* (order), CHKCI001 (genus), *Firmicutes* (phylum), *Clostridia* (class), and *Faecalibacterium* (genus). Moreover, *Bacteroides clarus* (species), *Clostridia* vadinBB60 group (family), *Clostridia* vadinBB60 group (genus), *Clostridia* vadinBB60 group (order), *Oscillospiraceae* (family), *Rikenellaceae* (family), *Alistipes* (genus), *Bacteroides* (genus), *Bacteroidaceae* (family), *Bacteroidia* (class), *Bacteroidota* (phylum), *Bacteroidales* (order) were enriched in the Control group.

### Correlation of cecal microbiota and growth performance

**Figure 4:**
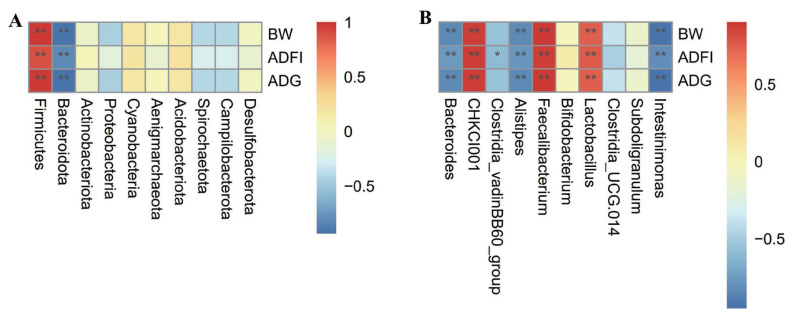
Correlation between cecal microbiota at phylum (A) or genus (B) level and growth performance of broilers. The magnitude of Spearman correlation coefficient is expressed by the change from red (positive correlation) to blue (negative correlation). * Indicates p<0.05. ** Indicates p<0.01.

The abundance of *Firmicutes* (phylum), CHKCI001 (genus), *Faecalibacterium* (genus), and *Lactobacillus* (genus) were all positively correlated with BW, ADFI and ADG, whereas the abundance of *Bacteroidota* (phylum), *Bacteroides* (genus), *Alistipes* (genus), and *Intestinimonas* (genus) were all negatively correlated with BW, ADFI, and ADG, respectively (Figure 4A, 4B). Besides, the abundance of *Clostridia* vadinBB60 group (genus) was negatively correlated with ADFI of broilers during the starter phase (Figure 4B).

### Functional prediction of cecal microbiota

**Figure 5:**
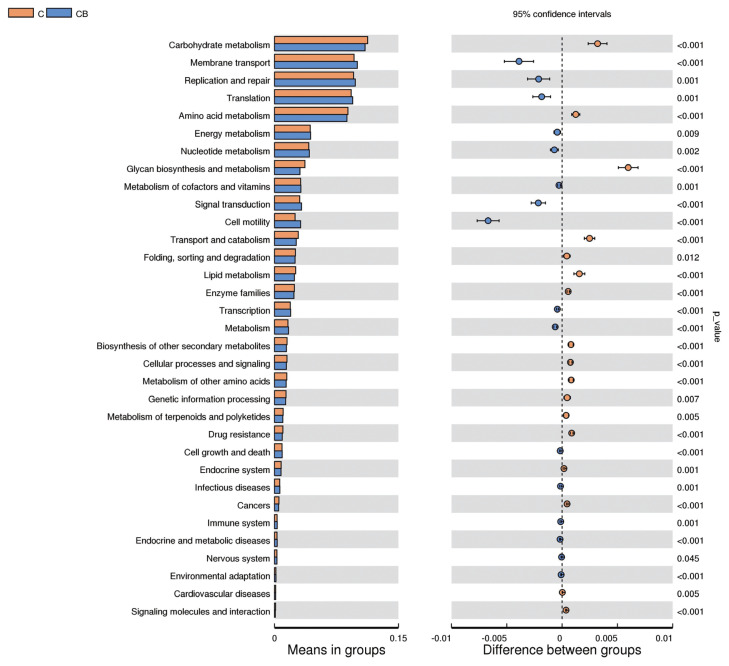
Differences in functional pathways. The functional pathways of cecal microorganisms were predicted using Tax4Fun. C, basal diet provided as control; CB, basal diet supplemented with 2×10^8^ CFU/kg of *C. butyricum* and 1×10^9^ CFU/kg of *B. subtilis*; CFU, colony forming units.

The presumptive biological function differences of the cecal microflora between the control group and the probiotic-supplemented group of broilers are shown in Figure 5.

At level 2, a total of 33 pathways with differences were found. Among them, the enrichment degree of carbohydrate metabolism, amino acid metabolism, glycan biosynthesis and metabolism, transport and catabolism, folding, sorting and degradation, lipid metabolism, enzyme families, biosynthesis of other secondary metabolites, cellular processes and signaling, metabolism of other amino acids, genetic information processing, metabolism of terpenoids and polyketides, drug resistance, endocrine system, cancers, cardiovascular diseases, and signaling molecules and interaction in the broiler chickens fed with *C. butyricum* and *B. subtilis* were significantly lower (p<0.05) than that of the control group. The functional pathway associated with membrane transport, replication and repair, translation, energy metabolism, nucleotide metabolism, metabolism of cofactors and vitamins, signal transduction, cell motility, transcription, metabolism, cell growth and death, infectious diseases, immune system, endocrine and metabolic diseases, nervous system, and environmental adaptation were increased (p<0.05) in the *C. butyricum* and *B. subtilis* group.

## DISCUSSION

### Compatibility of *Clostridium butyricum* and *Bacillus subtilis* on growth performance of broilers

Body weight, ADG, ADFI, and FCR are commonly used indicators to reflect the growth performance of poultry. Nowadays, research on the effect of *C. butyricum* and *B. subtilis* on the growth performance of broilers is mostly focused on the role of single bacterium, while the research on the effect of compatibility of the two bacteria is rare. Yu et al [18] found that addition of 1×10^9^ CFU/kg of *C. butyricum* had no significant effect on the ADG, ADFI, and FCR of Cobb broilers during the starter phase. However, Takahashi et al [5] reported that dietary addition of 2.5×10^8^ CFU/kg of *C. butyricum* MIYAIRI 588 significantly enhanced the ADG and ADFI of male Cobb and Ross 308 broilers during the starter phase, and decreased the FCR of male Cobb and Ross 308 broilers during the starter phase. Ma et al [1] found that adding 1×10^9^ CFU/kg of *B. subtilis* DSM32315 to diet significantly enhanced the BW of 28-day-old male Arbor Acres (AA) broilers, increased the ADG of starter broilers (1 to 28 days), but had no significant effect on the ADFI and FCR of broilers at this stage. Similarly, Mohamed et al [19] also observed that supplementation of 5×10^8^ CFU/kg of *B. subtilis* ATCC19659 significantly increased the BW of 21-day-old AA broilers and increased the ADG of broilers during the starter phase, but the ADFI and FCR of broilers at this stage were not affected. However, Wang et al [20] and Xu et al [21] respectively reported that adding 1×10^9^ CFU/kg of *B. subtilis* DSM29784 to diet did not affect the growth performance of Lingnan yellow broilers at the 1 to 21 day age stage, and adding 1.5×10^9^ CFU/kg of *B. subtilis* to diet also did not affect the growth performance of male Cobb 500 broilers during the starter phase. The results of this study showed that addition of 2×10^8^ CFU/kg of *C. butyricum* and 1×10^9^ CFU/kg of *B. subtilis* in diet significantly increased the BW of 21-day-old Ross 308 broilers, and enhanced the ADG and ADFI of starter broilers, but the FCR of broilers at this stage were not affected. Comparing the results of this experiment with those of previous studies, it was found that for the indicator of BW, the results of this experiment were consistent with the results of Ma et al [1] and Mohamed et al [19] (probiotics improved BW), but inconsistent with the results of Wang et al [20] and Xu et al [21] (probiotics had no effect on BW). Regarding the indicator of ADG, the results of this study were consistent with the results of Takahashi et al [5], Ma et al [1], and Mohamed et al [19] (probiotics increased ADG), but inconsistent with the results of Yu et al [18], Wang et al [20], and Xu et al [21] (probiotics had no effect on ADG). Regarding the indicator of ADFI, the results of this study were consistent only with the results of Takahashi et al [5] (probiotics increased ADFI), but inconsistent with the results of Yu et al [18], Ma et al [1], Mohamed et al [19], Wang et al [20], and Xu et al [21] (probiotics had no effect on ADFI). Regarding the indicator of FCR, the results of this study were consistent with all the studies (probiotics had no effect on FCR) except Takahashi et al [5] (probiotics decreased FCR). The discrepancy among these studies may be related to the differences in broiler breeds and ages, the types (including whether two probiotics were added simultaneously) and amounts of probiotics used, feeding management levels, and diet compositions in each study. The improved growth performance of broilers in the probiotic group observed in this experiment may be related to the following two aspects: on the one hand, *C. butyricum* or *B. subtilis* produced beneficial metabolites, such as butyric acid, vitamin B, digestive enzymes, etc., which promoted the digestion and absorption of nutrients [2,21]; on the other hand, the addition of *C. butyricum* and *B. subtilis* promoted the healthy intestinal microecological environment and improved the immunity of broilers [22,23].

### Compatibility of *Clostridium butyricum* and *Bacillus subtilis* on serum lipid of broilers

The lipids synthesized by the broiler liver need to be transported to other tissues through the blood, so the blood lipid content can reflect the metabolism of lipids in the body [24]. In this study, the concentrations of TG and TC in the serum of broilers in the probiotic group had no significant changes, indicating that the simultaneous addition of *C. butyricum* and *B. subtilis* had no effect on the stability of lipid metabolism in broilers under the conditions of this experiment. The homeostatic mechanism existing in broilers may be responsible for this result, but it needs to be further verified.

At present, the research on the effects of *C. butyricum* and *B. subtilis* on serum lipid in broilers mostly focused on the role of a single bacterium, but the research on the effects of compatibility was lacking. Liao et al [9] and Zhao et al [4] reported that both 2.5×10^8^ CFU/kg and 5×10^8^ CFU/kg supplementation levels of *C. butyricum* had no effect on the contents of TG and TC in the serum of 21-day-old male AA broiler chickens. However, Mohamed et al [6] showed that dietary supplementation of 5×10^8^ CFU/g of *B. subtilis* ATCC19659 significantly reduced the concentrations of TG and TC in the serum of 21-day-old AA broiler chickens, and pointed out the reason for this phenomenon may be that the addition of *B. subtilis* promotes the excretion of lipids from broiler feces and reduces the synthesis of lipids by inhibiting the lipid synthesis-related enzyme activity in broilers. The above results are not completely consistent, which may be due to the differences in the broiler breeds, the types and amounts of probiotics used, the diet compositions, and the feeding management levels.

### Compatibility of *Clostridium butyricum* and *Bacillus subtilis* on lipid synthesis-related enzyme activity of broilers

In the process of fatty acid synthesis, acetyl-CoA in the mitochondria is firstly transported to the cytosol by citric acid-pyruvate cycle, and then the acetyl-CoA is used as the raw material to synthesize malonyl-CoA, and the carbon chain is extended through the cycle reaction to obtain the synthesized fatty acid [25]. In this process, ACC, FAS, and NADP-ME all play an important role [26,27]. Since the liver is the main site for fatty acid synthesis in broilers [28], this experiment was carried out to measure the activities of lipid synthesis-related enzymes (including ACC, FAS, and NADP-ME) in the liver of broilers. It was found that the NADP-ME activity in the liver of broiler chickens was significantly increased after the addition of *C. butyricum* and *B. subtilis*, indicating that the addition of *C. butyricum* and *B. subtilis* can promote the production of reducednicotinamide-adenine dinucleotide phosphate in the liver of broilers, thus providing more energy for the de novo synthesis of fatty acids [28]. The reason for the increase in NADP-ME activity in the liver of broilers in the probiotic group may be that the addition of probiotics reduced the circulating factor ANGPTL4 which is recognized as an important mediator connecting intestinal microorganisms and host lipid metabolism [4]. However, this needs further verification.

At present, there are few studies on the effects of compatibility of *C. butyricum* and *B. subtilis* on the activities of enzymes related to lipid synthesis in the liver of broilers. However, it was found that 1×10^9^ CFU/kg of *C. butyricum* had no significant effect on the activities of FAS and ME in the liver of 21-day-old broilers [3] and on the activity of FAS enzymes in the liver of 42-day-old broilers [29]. Dietary supplementation of *C. butyricum* at a dose of 2.5×10^9^ CFU/kg significantly increased the expression of FAS mRNA in the liver of aged laying hens, but had no significant effect on the expression of ACC mRNA in the liver [30]. Besides, 4×10^11^ CFU/kg of *B. subtilis* significantly decreased the expression of FAS and ACCα mRNA in the liver of swine [31]. The above results are not completely consistent with the results of this experiment, which may be due to the differences in the types and levels of probiotics added, the types and ages of animals raised, diet composition, and feeding management levels.

### Compatibility of *Clostridium butyricum* and *Bacillus subtilis* on antioxidant status of broilers

Low levels of reactive oxygen species in cells are essential for normal metabolism, but their overproduction may induce oxidative stress in cells, leading to damage to DNA, proteins and lipids, with deleterious consequences for animal growth performance and product quality [32,33]. Therefore, the antioxidant capacity is closely related to the health of animals. Total antioxidant capacity and GSH-Px activity can reflect the antioxidant capacity, while MDA content can reflect the level of lipid peroxidation [34,35]. The unchanged T-AOC, GSH-Px activity, and MDA content in the serum and liver of birds in the probiotic group in this study indicated that simultaneous addition of *C. butyricum* and *B. subtilis* did not negatively affect the antioxidant status of 21-day-old broiler chickens. At present, results of studies on the effects of *C. butyricum* and *B. subtilis* on the antioxidant status of broilers are not consistent. Liao et al [9] reported that diet supplementation of 2.5×10^8^ CFU/kg of *C. butyricum* did not affect GSH-Px activity in the serum and liver and MDA content in the serum of 42-day-old male AA broilers, but significantly reduced MDA content in the liver. Yu et al [18] showed that dietary supplementation of 1×10^9^ CFU/kg of *C. butyricum* significantly enhanced GSH-Px activity (serum and liver) and decreased MDA content (serum and liver) of 21-day-old Cobb broilers, but had no effect on T-AOC (serum and liver). Bai et al [36] found that dietary supplementation of 2×10^10^ CFU/kg of *B. subtilis* fmbJ significantly enhanced the GSH-Px activity in the liver and significantly reduced the MDA content in the serum and liver of 42-day-old male AA broilers, but had no effect on the GSH-Px activity in the serum. Wang et al [20] showed that 1×10^9^ CFU/kg of *B. subtilis* DSM29784 significantly increased the serum GSH-Px activity of 63-day-old Lingnan yellow broilers, but the T-AOC and MDA content (serum) was not affected. Xu et al [21] found that dietary supplementation of 1.5×10^9^ CFU/kg of *B. subtilis* significantly increased GSH-Px activity (serum) and decreased MDA content (serum) of 42-day-old male Cobb 500 broiler chickens. Mohamed et al [19] showed that dietary supplementation of 5×10^8^ of *B. subtilis* ATCC19659 significantly increased serum T-AOC and decreased serum MDA content of 42-day-old AA broilers. The reason for the inconsistency of the above results may be due to the differences in the types and amounts of probiotics used, the animal breed and age, the diet composition, and the feeding management levels in each experiment. The reason why *C. butyricum* and *B. subtilis* mentioned above improved the antioxidant capacity of broilers is probably related to the regulation of Nrf2 signaling pathway which is closely related to antioxidant capacity [2,37].

### Compatibility of *Clostridium butyricum* and *Bacillus subtilis* on cecal microflora of broilers

The large number of microorganisms present in the cecum of poultry is of great significance for promoting the digestion and absorption of nutrients in poultry and maintaining the stability of the intestinal microecology of poultry [1]. In recent years, probiotics as a microbial regulator has attracted more and more attention with the prohibition of antibiotics. At present, research on the effects of *C. butyricum* and *B. subtilis* on cecal microflora of broilers are mostly focused on the role of single bacterium, but the information on the effects of compatibility is lacking. Yu et al [18] found that adding 1×10^9^ CFU/kg of *C. butyricum* to diet significantly reduced the abundance of *Alistipes* in the cecum of 21-day-old Cobb broilers, but had no effect on the abundance of *Bacteroides* and *Faccalibacterium* and the Chao 1 index of microorganisms in the cecum. Jacquier et al [38] reported that *B. subtilis* 29784 at a supplemental level of 1×10^8^ CFU/kg did not affect the Shannon index of cecal microbiota in 42-day-old Cobb 500 broilers, but significantly reduced the abundance of cecal *Bacteroides*. Ma et al [1] pointed out that *Firmicutes* and *Bacteroidota* accounted for more than 95% of the whole phyla in the cecum of 42-day-old broilers. Adding 1×10^9^ CFU/kg of *B. subtilis* DSM32315 to diet had no significant effect on the alpha diversity (including Shannon, Simpson, Chao1, etc.) of cecal microbiota in 42-day-old AA broilers, but enhanced the abundance of *Firmicutes* and *Clostridia*, and reduced the abundance of *Bacteroidota* in the cecum of broilers. Similar results were also obtained by Mohamed et al [19] by feeding *B. subtilis* ATCC19659 to AA broilers. Zhang et al [39] showed that dietary supplementation of 1.5×10^10^ to 2.25×10^10^ CFU/kg of *B. subtilis* (LIFEGUFS-S200) did not affect the alpha diversity (including Shannon, Simpson, Chao1, etc.) of cecal microorganisms in 42-day-old AA broilers, but enhanced the abundance of *Faecalibacterium* and *CHKCI001* in the cecum of broilers. The above results regarding the variation in cecal microbiota composition of broilers were similar to the results of this study, but the results regarding the Shannon and Simpson index of cecal microbiota were inconsistent with the results of this study. Shannon and Simpson index mainly reflects the diversity and evenness of species distribution in the cecal microbial community of broilers. In general, the larger the Shannon and Simpson index, the higher the community diversity and the more even the species distribution. The compatibility of *C. butyricum* and *B. subtilis* significantly decreased the Shannon and Simpson index of cecal microbiota in this experiment, indicating that compatibility of *C. butyricum* and *B. subtilis* reduced the microbial community diversity and species distribution uniformity in broilers, but the reasons need to be further explored.

In addition, this study also found that the difference of cecal microflora at the phylum and genus level of 21-day-old broilers caused by the compatibility of *C. butyricum* and *B. subtilis* was correlated with the growth performance. The results of the positive correlation between the abundance of *Firmicutes* and the growth performance of broilers and the negative correlation between the abundance of *Bacteroidota* and the growth performance of broilers were consistent with the results of Ley et al [40] and Ley et al [41], and also consistent with the results found in this study that the compatibility of *C. butyricum* and *B. subtilis* significantly enhanced the growth performance of broiler chickens. These results indicated that compatibility of *C. butyricum* and *B. subtilis* could improve the growth performance of 21-day-old broilers by changing the microflora in the cecum. However, the correlation between the cecal microbiota at genus level and the growth performance of 21-day-old broilers is not completely consistent with the research results of Gong et al [42], Hu et al [43] and Zhang et al [39], which needs to be further verified by *in vivo* and *in vitro* studies.

The changes of host intestinal microecological environment caused by dietary intervention in broiler chickens might lead to changes in some specific functional pathways [44]. To infer the effect of *C. butyricum* and *B. subtilis* on the functional pathway of cecal microflora in broilers, Tax4Fun analysis was carried out in this study. The results showed that simultaneous supplementation of *C. butyricum* and *B. subtilis* had significant effects on 33 different functional pathways such as lipid metabolism, carbohydrate metabolism, and energy metabolism in broilers. This is inconsistent with the results of Ma et al [1] and Zhang et al [39] who found that *B. subtilis* had little effect on the predicted functions of cecal microbiota in broilers. The reasons for the inconsistent results may be due to the differences in animal breeds and ages, diet composition, management levels, types and amounts of probiotics used, and the methods for functional pathways prediction of cecal flora. Of course, the current analysis of functional pathways differences in this study is predictive, and further studies are needed to confirm the specific functional pathways of different microbes.

## CONCLUSION

Simultaneous supplementation of *C. butyricum* and *B. subtilis* could modify the diversity and composition of cecal bacteria in broilers during the starter phase, causing changes in 33 different functional pathways such as lipid metabolism, carbohydrate metabolism, and energy metabolism, thereby promoting the growth performance and the lipid synthesis-related enzyme activity in the liver of broilers. However, these need to be further verified. In addition, simultaneous addition of *C. butyricum* and *B. subtilis* did not affect the serum lipids and antioxidant status of broilers during the starter phase.

## Figures and Tables

**Table 1:** Composition and nutrient levels of basal diet (air-dry basis, %)

Items	Content
Ingredients	
Corn	35.60
Wheat	25.00
Extruded soybean meal (43.2% CP)	26.20
Cottonseed meal	4.00
Corn gluten meal	1.00
Hydrolyzed feather meal	1.00
CaHPO_4_	0.90
Limestone	1.70
Soy oil	2.50
L-Lys (70%)	0.70
DL-Met	0.25
L-Thr	0.15
Premix^1)^	1.00
Total	100.00
Nutrient levels, analyzed	
ME, calculated (kcal/kg)	2,948
CP	21.98
Calcium	0.94
Total phosphorus	0.65
Lys	1.41
Met	0.57
Met+Cys	0.91
Thr	0.94

CP, crude protein; ME, metabolizable energy.

1) Supplied per kilogram of diet: vitamin A, 8,000 IU; cholecalciferol, 1,000 IU; vitamin E, 20 IU; vitamin K_3_, 0.50 mg; thiamin, 2 mg; riboflavin, 8 mg; pantothenic acid, 10 mg; cobalamin, 0.010 mg; niacin, 35 mg; biotin, 0.18 mg; folic acid, 0.55 mg; Mn, 100 mg; Fe, 80 mg; Zn, 80 mg; Cu, 8 mg; I, 0.7 mg; and Se, 0.3 mg.

**Table 2:** Compatibility of *Clostridium butyricum* and *Bacillus subtilis* on growth performance of broilers during the starter phase^1)^

Items	C^2)^	CB^2)^	p-value
Body weight (g)	852.86±8.08^b^	931.87±12.67^a^	<0.001
Average daily gain (g/d)	49.14±0.39^b^	53.54±0.52^a^	<0.001
Average daily feed intake (g/d)	38.69±0.38^b^	42.44±0.60^a^	<0.001
Feed conversion rate (g/g)	1.27±0.01	1.26±0.01	0.502

Results are expressed as mean±standard error.

1) Data are means for 6 replicates of 50 chicks.

2) C, control group; CB, probiotic group (including 2×10^8^ CFU/kg of *C. butyricum* and 1×10^9^ CFU/kg of *B. subtilis*).

a,b Means within a row with different letters differ significantly (p<0.05).

**Table 3:** Compatibility of *Clostridium butyricum* and *Bacillus subtilis* on serum lipid of broilers at 21 days of age^1)^

Items	C^2)^	CB^2)^	p-value
Triglyceride (mmol/L)	0.99±0.06	1.10±0.15	0.495
Total cholesterol (mmol/L)	4.31±0.42	4.11±0.60	0.799

Results are expressed as mean±standard error.

1) Data are means for 6 replicates of 1 chick.

2) C, control group; CB, probiotic group (including 2×10^8^ CFU/kg of *C. butyricum* and 1×10^9^ CFU/kg of *B. subtilis*).

**Table 4:** Compatibility of *Clostridium butyricum* and *Bacillus subtilis* on lipid synthesis-related enzyme activity in the liver of broilers at 21 days of age^1)^

Items	C^2)^	CB^2)^	p-value
Fatty acid synthase (nmol/min/mg prot)	3.20±0.18	3.40±0.22	0.499
NADP-malic enzyme (nmol/min/mg prot)	22.44±1.75^b^	30.27±2.18^a^	0.034
Acetyl-CoA carboxylase (μmol/h/mg prot)	13.44±0.30	13.37±0.29	0.864

Results are expressed as mean±standard error.

1) Data are means for 6 replicates of 1 chick.

2) C, control group; CB, probiotic group (including 2×10^8^ CFU/kg of *C. butyricum* and 1×10^9^ CFU/kg of *B. subtilis*).

a,b Means within a row with different letters differ significantly (p<0.05).

**Table 5:** Compatibility of *Clostridium butyricum* and *Bacillus subtilis* on antioxidant status of broilers at 21 days of age^1)^

Items	C^2)^	CB^2)^	p-value
Serum			
Total antioxidant capacity (U/mL)	11.94±0.76	14.03±1.10	0.157
Glutathione peroxidase (U/mL)	2,638.3±201.5	3,003.5±241.8	0.273
Malondialdehyde (nmol/mL)	4.12±0.26	3.90±0.24	0.533
Liver			
Total antioxidant capacity (U/mg prot)	0.89±0.09	0.68±0.07	0.105
Glutathione peroxidase (U/mg prot)	33.32±1.00	33.82±0.67	0.697
Malondialdehyde (nmol/mg prot)	4.46±0.52	3.98±0.32	0.451

Results are expressed as mean±standard error.

1) Data are means for 6 replicates of 1 chick.

2) C, control group; CB, probiotic group (including 2×10^8^ CFU/kg of *C. butyricum* and 1×10^9^ CFU/kg of *B. subtilis*).

**Table 6:** Compatibility of *Clostridium butyricum* and *Bacillus subtilis* on alpha diversity of cecal microbiota of broilers at 21 days of age^1)^

Items	C^2)^	CB^2)^	p-value
Chao1	571.55±29.60	553.48±36.50	0.709
Observed_otus	570.67±29.47	552.83±36.29	0.711
Shannon	7.10±0.08^a^	6.60±0.12^b^	0.005
Simpson	0.98±0.00^a^	0.96±0.00^b^	0.004

Results are expressed as mean±standard error.

1) Data are means for 6 replicates of 1 chick.

2) C, control group; CB, probiotic group (including 2×10^8^ CFU/kg of *C. butyricum* and 1×10^9^ CFU/kg of *B. subtilis*).

a,b Means within a row with different letters differ significantly (p<0.05).
